# Performance of Bimanual Finger Coordination Tasks in Speakers Who Stutter

**DOI:** 10.3389/fpsyg.2021.679607

**Published:** 2021-09-23

**Authors:** Akira Toyomura, Tetsunoshin Fujii, Paul F. Sowman

**Affiliations:** ^1^Graduate School of Health Sciences, Gunma University, Maebashi, Japan; ^2^Research Center for Advanced Technologies, Tokyo Denki University, Inzai, Japan; ^3^Department of Psychology, Hokkaido University, Sapporo, Japan; ^4^School of Psychological Sciences, Faculty of Medicine, Health and Human Sciences, Macquarie University, Sydney, NSW, Australia

**Keywords:** stuttering, finger movement, mirror and parallel tapping, motor dexterity, timing control, basal ganglia, cerebellum, supplementary motor area

## Abstract

Stuttering is a neurodevelopmental speech disorder characterized by the symptoms of speech repetition, prolongation, and blocking. Stuttering-related dysfluency can be transiently alleviated by providing an external timing signal such as a metronome or the voice of another person. Therefore, the existence of a core motor timing deficit in stuttering has been speculated. If this is the case, then motoric behaviors other than speech should be disrupted in stuttering. This study examined motoric performance on four complex bimanual tasks in 37 adults who stutter and 31 fluent controls. Two tasks utilized bimanual rotation to examine motor dexterity, and two tasks used the bimanual mirror and parallel tapping movements to examine timing control ability. Video-based analyses were conducted to determine performance accuracy and speed. The results showed that individuals who stutter performed worse than fluent speakers on tapping tasks but not on bimanual rotation tasks. These results suggest stuttering is associated with timing control for general motor behavior.

## Introduction

Stuttering is a speech fluency disorder. Most developmental stuttering cases have their onset between 2 and 5 years of age, and the population incidence ranges from 1 to 11% (Craig et al., 2002; McLeod and Harrison, 2009; Boyle et al., 2011; Reilly et al., 2013). Sixty to eighty percent of developmental stuttering cases recover without intervention (Kefalianos et al., 2017; Shimada et al., 2018); the remainder will often continue to experience lifelong speech disfluency. It is estimated that more than 10 million people across the world stutter; however, neither definite causes for stuttering nor foolproof treatments are known. Recent biological studies on stuttering postulate a complex neurodevelopmental disorder resulting from interactions between the genes and the environment (Ooki, 2005; Rautakoski et al., 2012; Frigerio-Domingues and Drayna, 2017), which neurological studies suggest manifest as altered function and structure of the brain.

One promising theory posits that stuttering results from a deficit in speech timing control (Van Riper, 1982; Etchell et al., 2014a). This notion accounts for the well-known phenomenon that, in stuttering, dysfluency can be temporarily suppressed by providing external timing cues; for example, speech synchronized to the beat of a metronome is generally devoid of dysfluencies. Furthermore, choral reading, where other cooperating speakers in the chorus provide timing cues for speech rhythm, enhances fluency in people who stutter.

Fluency-induction, *via* the provision of external timing stimuli, has led to speculation that causative brain regions in stuttering are likely related to timing functions. Specifically, the basal ganglia degeneration that occurs in Parkinson's disease (PD) leads to movement quality deficits that are ameliorated when external timing stimuli are provided, which has led to speculation that stuttering might also be associated with basal ganglia dysfunction (Alm, 2004; Etchell et al., 2014a). Several studies have reported altered structure or function of the basal ganglia in stuttering participants compared with fluent controls. Such alterations include less metabolic activity (e.g., Wu et al., 1995; Toyomura et al., 2011, 2015; Connally et al., 2018), altered connectivity (Lu et al., 2010b; Chang and Zhu, 2013; Qiao et al., 2017), and reduced (Beal et al., 2013; Foundas et al., 2013; Sowman et al., 2017) or increased gray matter volume (Lu et al., 2010b) of the basal ganglia.

If impaired timing control due to malfunction in large-scale brain networks causes stuttering, behavioral manifestations of this outside the domain of speech might also be expected. There are studies reporting specific motor performance decrements in finger movement tasks in individuals who stutter (Webster, 1986, 1988, 1990; Zelaznik et al., 1997; Smits-Bandstra et al., 2006a,b; Choo et al., 2016). For example, Webster (1990) showed that tapping rates of a bimanual-asymmetrical tapping task were significantly slower in adults who stutter than in fluent controls. Webster (1988) showed that adults who stuttered were slower on a bimanual handwriting task, made more mistakes, and produced a poorer quality output than the fluent controls they were compared with. Smits-Bandstra et al. (2006a,b) investigated the speech and non-speech sequence skill learning in adults who stutter and fluent speakers and reported that the finger-tapping task induced significantly poorer performance in the stuttering group than the control group. Falk et al. (2015) investigated timing control in finger tapping to periodic tone sequences and a musical beat and showed that children and adolescents who stutter showed poorer synchronization to both metronome and musical stimuli than fluent controls. Conversely, some recent studies have reported no differences in finger sequence learning (Korzeczek et al., 2020) or manual tasks using the Purdue Pegboard Test (Werle et al., 2019).

Recently, novel bimanual coordination tasks, e.g., in-phase and antiphase finger movement paradigms (Wu et al., 2010; Aramaki et al., 2011; Buchanan et al., 2017; Vaz et al., 2017), have been widely adopted in neuroimaging and behavioral studies of motor control. Because such paradigms require precise synchronization of both hands, they tap into “timing control” aspects of motor production. Using such tasks, Wu et al. (2010) showed that patients with PD performed comparatively poorly in the antiphase task and at the same time exhibited less activity in the basal ganglia and supplementary motor areas (Wu et al., 2010). Bimanual coordination tasks have also been used to investigate “motor dexterity” in various neurological diseases (Midorikawa et al., 2008). In their study, Midorikawa et al. (2008) used a finger movement task where participants were required to rotate a finger pair while keeping the remaining fingers connected and fixed at the ball. They showed that this task could distinguish between controls and patients with schizophrenia. The task used in Midorikawa et al. (2008) is similar to one of the constituent tasks of the Dow-Moruzzi motor battery, which has been used to estimate cerebellar dysfunction (Dow and Moruzzi, 1958; Fawcett et al., 1996; Ramus et al., 2003). This battery consists of several tests, including bimanual coordination, bead threading, postural stability, and time estimation. Although these tasks are potent experimental paradigms for investigating motor function in patients and controls, such methods have not yet been applied to a cohort of people who stutter.

In this study, we tested the finger movement tasks described above on adults who stutter to investigate their motor dexterity and timing control ability. Our goal was to extend the understanding of motor control in stuttering. The two bimanual tasks, namely, the “motor dexterity task” and the “timing control task,” referred to above were adopted for this experiment. In the motor dexterity task, participants connected the fingers of both hands and were required to perform complex finger movements as instructed. Precise coordination of both hands is required; hence, this task is most associated with “motor dexterity.” The timing control task requires in-phase (Mirror) and antiphase (Parallel) tapping (e.g., Aramaki et al., 2011). Participants move the fingers of both hands independently and separately, and hence, timing control is of primary importance in order that the required phase relationship between hands is maintained. If a deficit in timing control is the cause of stuttering, these experiments could separate that from a general motor control deficit, which would also be evident in other domains of motor control, e.g., dexterity.

## Methods

### Participants

Sixty-eight adults participated in this study. Thirty-seven of these were adults who stutter (seven women, aged 19–52 years with a mean age of 33.4 years, *SD* = 10.0, 35 right-handed and two left-handed), and 31 were fluent controls (nine women, aged 20–56 years with a mean age of 28.8 years, *SD* = 9.7, 29 right-handed, one left-handed, and one ambidextrous). The age of the two groups was not significantly different [*t*_(66)_ = 1.87, *p* = 0.07]. However, because of a trend toward a significant difference, age was entered as a covariate in the analysis of covariance (ANCOVA) (as shown in section Analysis). All participants were native speakers of Japanese. Adults who stutter were recruited from stuttering self-help communities. Participants in the control group did not stutter. Before or after the experimental tasks, participants engaged in a conversation task with the experimenters in front of a video camera. Stuttering severity was evaluated as percent syllables stuttered (% SS) based on the video-recorded speech samples. The % SS ranged from 0 to 24.6% (mean = 3.4, *SD* = 4.6, <1% SS = 11 participants, 1% SS or more and <5% SS = 18 participants, 5% SS or more and <10% SS = 6 participants, 10% SS or more and <30% SS = 2 participants). Among the participants who stutter, one did not stutter (0% SS) in front of the video camera. However, that participant disclosed that they generally stuttered in difficult situations and so was included in further analyses. The ethics committees of Tokyo Denki University and Gunma University approved this study. Written informed consent was obtained from all participants according to the Declaration of Helsinki.

### Experimental Paradigms

Participants performed the following finger movement tasks:

#### Rotation Task 1

**Figure 1:**
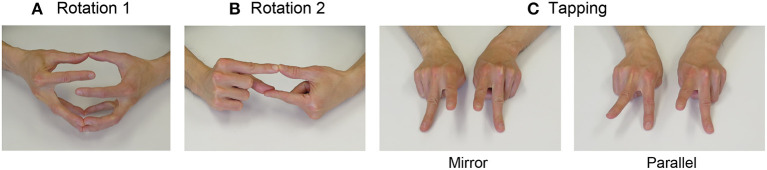
Snapshots of each finger movement task. **(A)** Rotation task 1. Both hands were connected at the ball of each finger. Participants were then instructed to disconnect one pair of fingers and rotate them five times without separating the other finger pairs. Subsequently, in a similar manner, they rotated the pair five times in the opposite direction (for a total of 10 rotations). They were instructed to perform this movement as fast as possible, without disconnecting the other pairs of fingers. The picture example shows a pair of index fingers. They were instructed to make 10 rotations for each pair of thumbs, index, middle, annular, and fifth fingers. **(B)** Rotation task 2. The right index finger and left thumb were connected at the ball, and similarly, the left index finger and right thumb were connected at the ball. The other fingers were closed. First, one lower index finger/thumb pair was disconnected, and rotated upward, and was then connected again. Subsequently, the other index finger/thumb pair located below was disconnected, and was rotated upward, and connected again. Participants were instructed to perform this movement for 15 s as fast as possible. The same procedure was applied to all of the four pairs (index finger and thumb, middle finger and thumb, annular finger and thumb, and fifth finger and thumb). **(C)** Tapping task. In the Mirror task, the two index fingers of both hands were raised, and subsequently, the index fingers were lowered whilst simultaneously raising the middle fingers. In the Parallel task, the same movement was performed with the pair of the right index and left middle fingers, and the pair of the left index and right middle fingers. Participants were instructed to perform these movements for 30 s as fast as possible.

Both hands were connected at the ball of each finger. Participants were then instructed to disconnect one pair of fingers and rotate them five times without separating the other finger pairs. Subsequently, in a similar manner, they rotated the pair five times in the opposite direction. Therefore, there were 10 rotations in total. Participants were instructed to perform these rotations as fast as possible, without disconnecting the other finger pairs. They were instructed to make 10 rotations for each pair of thumbs, index, middle, annular, and fifth fingers. The picture example (Figure 1A) shows a pair of index fingers.

#### Rotation Task 2

The right index finger and left thumb were connected at the ball, and similarly, the left index finger and the right thumb were connected at the ball. The other fingers were closed. First, one lower pair of index finger and thumb was disconnected, rotated upward, and connected again. Subsequently, the other index finger and thumb pair located below was disconnected, rotated upward, and connected again. Participants were instructed to perform this movement for 15 s as fast as possible. The same procedure was applied to all four pairs (1. index finger and thumb; 2. middle finger and thumb; 3. annular finger and thumb; 4. fifth finger and thumb). The picture example (Figure 1B) shows a pair of index fingers and thumb. In the pilot experiment, we found that this movement was difficult for some participants, and they could not perform it for a long time. Therefore, we set this task length as 15 s and counted the number of times the task was correctly performed.

#### Tapping Task

Two kinds of finger tapping tasks, bimanual Mirror and Parallel tasks, were used. The Mirror task corresponds to the in-phase task, and the Parallel task corresponds to the antiphase task, the terms used in previous studies (Wu et al., 2010). Index and middle fingers of both hands were extended on the table, and the other fingers were flexed (Figure 1C). In the Mirror task, the two index fingers were raised, and subsequently, the index fingers were lowered down while simultaneously raising the middle fingers. In the Parallel task, the same movement was performed with the pair of right-index and left-middle fingers and a pair of left-index and right-middle fingers. Therefore, the participants were required to control bimanual movement timing and match the phase difference of the two hands (0 degrees in the Mirror task and 180 degrees in the Parallel task). Participants were instructed to perform these movements for 30 s as fast as possible.

All participants performed all tasks. We compared the performance between groups rather than between tasks. Participants performed the tasks in the following order: rotation 1, rotation 2, and tapping. Before the experiment, participants were presented with examples of each task by the experimenters and practiced each task in advance. Their behavior was video recorded for analysis.

### Analysis

The performance of participants was analyzed based on video recording. An independent person analyzed each video based on the criteria described in the following paragraphs. This rater was blind to whether the individual video data was from a person who stutters or not. During the analysis, the videos were played in slow motion when required. Sessions, where participants did not correctly follow the instructions, were excluded from the analysis. In addition, if the video was difficult to analyze because of the shooting angle, the corresponding sessions were also excluded. The performance in each task was measured as per the following procedures.

#### Rotation Task 1

The time that each participant took to complete the 10 rotations was measured for each pair of fingers. If the video image showed that the participant did not rotate the required number of times, the time was corrected based on the times of actual rotation. For example, a certain participant rotated only four times in each direction (eight times in sum), and it took 5 s; in this case, the time of 5 s was multiplied by 10/8 and the corrected time was 6.25 s. Similarly, if the participant rotated more than five times, the time was corrected in a similar manner.

#### Rotation Task 2

The number of times the participant correctly performed the required rotation in 15 s was measured. One performance was defined as a series of actions consisting of the participant disconnecting, rotating, and then reconnected the finger pair. If the participant disconnected other pairs of fingers, the rotation during that time was not counted.

#### Tapping Task

The number of times the participant correctly performed a tap in 30 s was counted. One complete tap was defined as the raising of the fingers of both hands simultaneously. If the video image showed that the periodic movement was broken, such as a nonparallel movement during the Parallel task, taps during the broken period were not counted.

#### Statistics

Two-way repeated measures ANCOVAs with the group as a between-participant factor and condition as a within-participant factor were conducted for the three tasks. Participant age was treated as a covariate. The factor of the group had two levels (adults who stutter and control) in all tasks. The factor of condition had five levels in Rotation task 1 (pairs of thumbs, index fingers, middle fingers, annular fingers, and fifth fingers), four levels in Rotation task 2 (pairs of index finger and thumb, middle finger and thumb, annular finger and thumb, and fifth finger and thumb), and two levels in the Tapping task (Mirror and Parallel). When the main effect of groups was significant, *post-hoc* comparisons were performed using one-way ANCOVA with a factor of group (adults who stutter and controls) and Bonferroni correction. This *post-hoc* was performed to investigate performance differences between groups within each finger combination. Correlation analyses were conducted to investigate the relation between stuttering frequency and behavioral performance in all conditions (Rotation task 1, Rotation task 2, and Tapping task).

To determine the measurement reliability of the analysis, a second independent evaluator analyzed the data for 20 participants (a random selection of 10 individuals who stutter and 10 fluent controls; 30% of the data). This rater was also blind to whether the individual video data was from a person who stutters or not, and to the scores of the first rater. Interrater reliability was calculated as follows: First, we calculated the congruent values (in the amount of time, number of times, etc.) and the incongruent values (difference between the two). The congruent value was divided by the congruent value plus the incongruent value. For example, in the Tapping task, if an evaluator extracts 50 successful taps and another evaluator extracts 48 successful taps from a video data, the agreement rate is calculated as 48/(48 + 2) × 100 = 96%. Measurements were pooled for 20 participants in each task. Interrater correlation between measurements was calculated. Differences between raters were analyzed with ANOVA.

## Results

### Exclusion of the Data

In the stuttering group, seven videos or 6.3% of the data (five for Rotation 1 and two for Rotation 2 tasks) were excluded from the analysis because of the participant not following instructions and/or the video angles. In the control group, two or 2.2% of the data (one for Rotation 1 and one for Rotation 2 tasks) were excluded for the same reasons.

### Interrater Reliability

For Rotation task 1, interrater reliability between the two independent evaluators was 99.1% for the pair of thumbs, 94.9% for the index fingers, 96.9% for the middle fingers, 96.9% for the annular fingers, and 94.6% for the fifth fingers. Interrater correlations between the successful repetitions counts extracted by the two evaluators were 0.990, 0.961, 0.966, 0.997, and 0.974, respectively, and a two-way repeated measures ANOVA with factors of raters and pairs showed no significant main effect of raters [*F*_(1,38)_ = 0.020, *p* = 0.889, partial η^2^ = 0.001].

For Rotation task 2, the reliability was 98.9% for the pair of index finger and thumb, 99.6% for the pair of middle finger and thumb, 98.3% for the pair of annular finger and thumb, and 98.3% for the pair of fifth finger and thumb. Interrater correlation between the successful repetitions counts for the two evaluators were 0.999, 1.000, 0.997, and 0.996, respectively, and a two-way repeated measures ANOVA with factors of raters and pairs showed no significant main effect of raters [*F*_(1,38)_ = 0.001, *p* = 0.970, partial η^2^ = 0.000].

Similarly, in the Tapping task, the reliability was 98.9% for the Mirror task and 98.1% for the Parallel task. The interrater correlation between the successful repetitions counts extracted by the two evaluators was 0.998 for both tasks, and a two-way repeated measures ANOVA showed no significant main effect of raters [*F*_(1,38)_ = 0.000, *p* = 0.987, partial η^2^ = 0.000].

### Rotation Task 1

**Figure 2:**
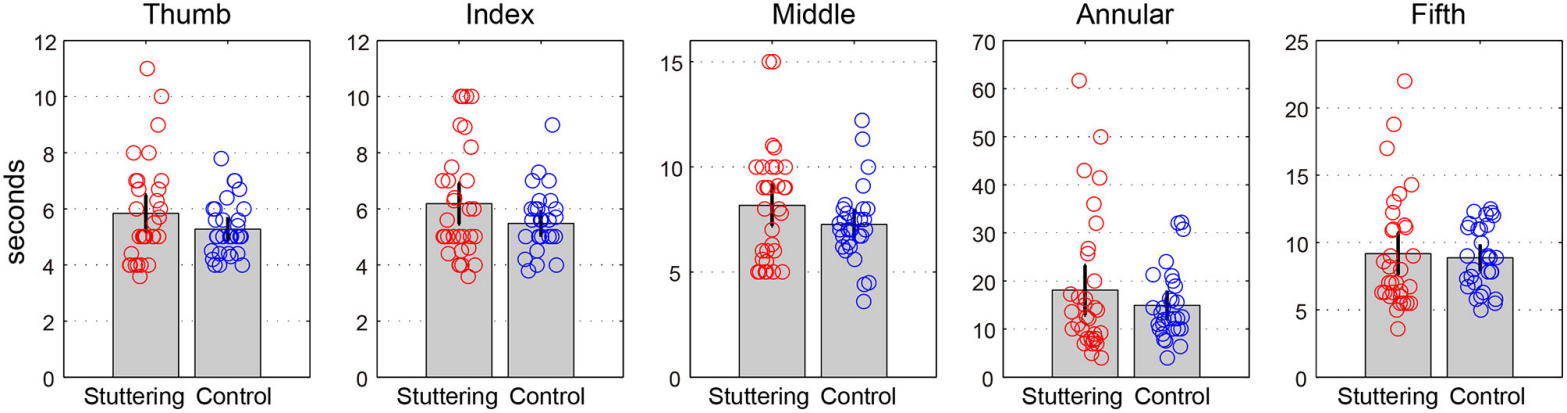
Rotation task 1. The y-axis represents the amount of time (s) for the 10 rotations in each finger pair in s. Error bars represent 95% confidence intervals.

Table 1 and Figure 2 show the time (in s) for the 10 rotations in each finger pair. A two-way repeated measures ANCOVA with factors of groups (adults who stutter and controls) and conditions (pairs of thumbs, index fingers, middle fingers, annular fingers, and fifth fingers) did not show any significant difference between groups [*F*_(1,59)_ = 0.627, *p* = 0.432, partial η^2^ = 0.011] or conditions [*F*_(1.1,67.7)_ = 0.239, *p* = 0.660, partial η^2^ = 0.004, Greenhouse-Geisser corrected]. There was no significant interaction between groups and conditions [*F*_(1.1,67.7)_ = 0.244, *p* = 0.656, partial η^2^ = 0.004, Greenhouse-Geisser corrected]. Adults who stutter did not show significant correlations between percent stuttered syllables and performance in any condition (Thumb: *r* = −0.064, *p* = 0.737; Index: *r* = −0.005, *p* = 0.981; Middle: *r* = −0.216, *p* = 0.252; Annular: *r* = −0.220, *p* = 0.242; Fifth: *r* = 0.015, *p* = 0.939).

**Table 1:** Rotation task 1.

	**Adults who stutter**	**Controls**
Thumb	5.8 ± 1.8	5.3 ± 1.0
Index	6.2 ± 2.0	5.5 ± 1.1
Middle	8.2 ± 2.6	7.3 ± 1.8
Annular	18.1 ± 14.1	14.9 ± 7.2
Fifth	9.2 ± 4.3	8.9 ± 2.3

*The numbers shown are in seconds (mean ± SD) for the 10 rotations of each finger pair*.

### Rotation Task 2

**Figure 3:**
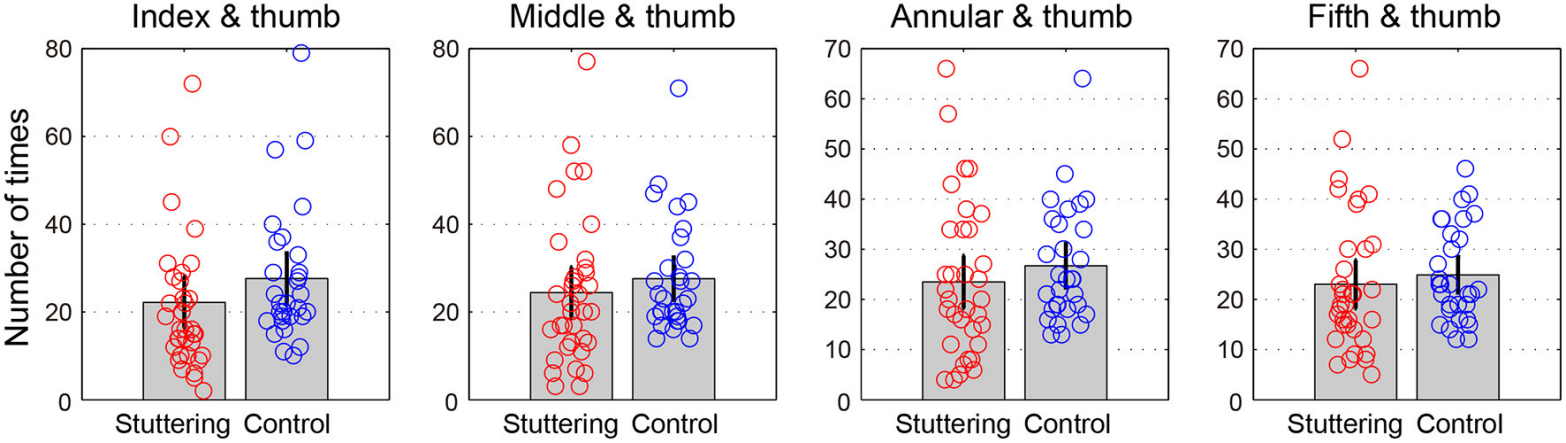
Rotation task 2. The y-axis represents the number of times performed in 15 s for each finger pair. Error bars represent 95% confidence intervals.

Table 2 and Figure 3 show the number of times successful rotations were performed in 15 s for each finger pair. A two-way repeated measures ANCOVA with factors of groups (adults who stutter and controls) and conditions (pairs of index finger and thumb, middle finger and thumb, annular finger and thumb, and fifth finger and thumb) did not show a significant difference between groups [*F*_(1,62)_ = 0.619, *p* = 0.434, partial η^2^ = 0.010] or conditions [*F*_(2.0,122.5)_ = 2.568, *p* = 0.081, partial η^2^ = 0.040, Greenhouse-Geisser corrected]. There was no significant interaction between groups and conditions [*F*_(2.0,122.5)_ = 1.751, *p* = 0.178, partial η^2^ = 0.027, Greenhouse-Geisser corrected]. Adults who stutter did not show significant correlations between percent stuttered syllables and performance in any condition (Index finger and thumb: *r* = −0.191, *p* = 0.287; Middle finger and thumb: *r* = −0.207, *p* = 0.248; Annular finger and thumb: *r* = −0.202, *p* = 0.260; Fifth finger and thumb: *r* = −0.224, *p* = 0.210).

**Table 2:** Rotation task 2.

	**Adults who stutter**	**Controls**
Index and thumb	22.2 ± 17.0	27.6 ± 15.2
Middle and thumb	24.4 ± 16.6	27.6 ± 12.8
Annular and thumb	23.5 ± 15.1	26.7 ± 11.4
Fifth and thumb	23.0 ± 13.8	24.9 ± 9.3

*Each shows the number of successful rotations performed (mean ±SD) in 15 s for each finger pair*.

### Tapping Task

**Figure 4:**
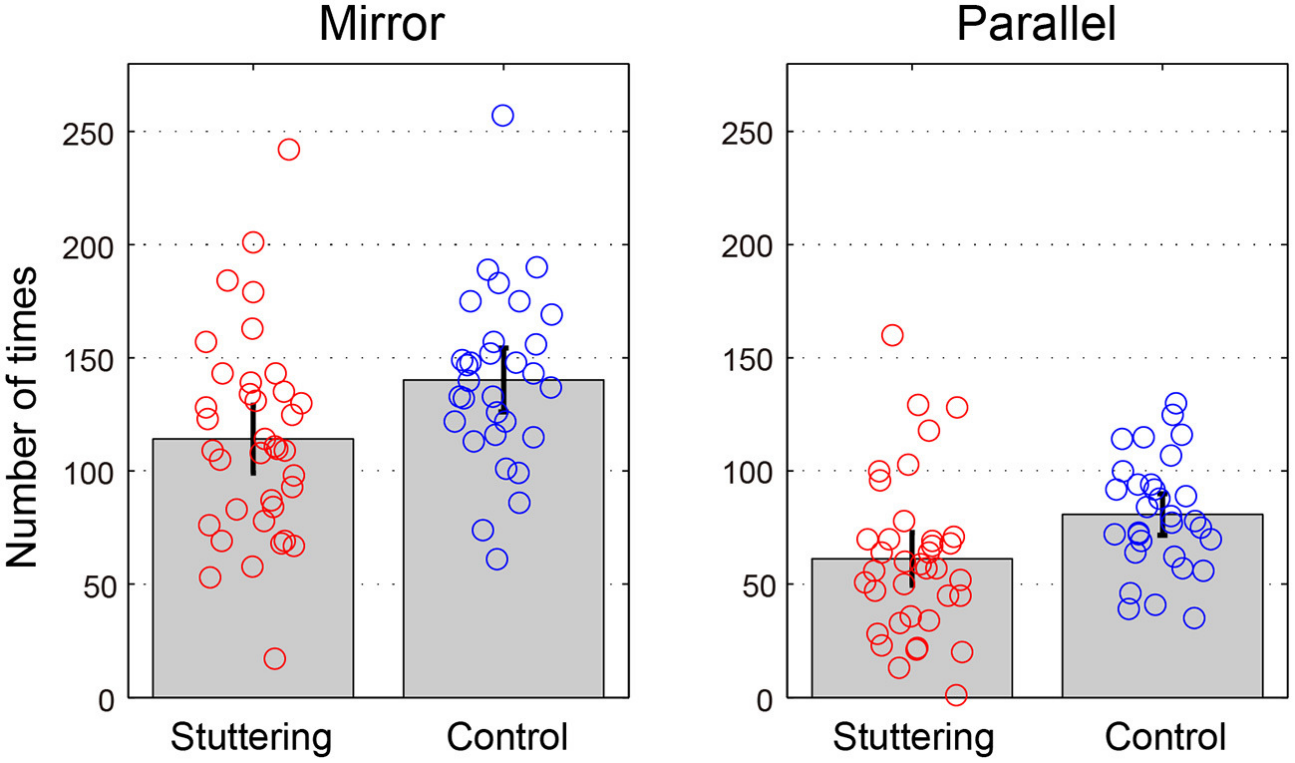
Tapping task. The y-axis represents the number of times performed in 30 s for each task. Error bars represent 95% confidence intervals.

Table 3 and Figure 4 show the number of successful taps performed in 30 s for each task. A two-way repeated measures ANCOVA with factors of groups (adults who stutter and controls) and conditions (Mirror and Parallel) showed a significant difference between groups [*F*_(1,65)_ = 5.286, *p* = 0.025, partial η^2^ = 0.075] and conditions [*F*_(1,65)_ = 9.591, *p* = 0.003, partial η^2^ = 0.129]. There was no significant interaction between groups and conditions [*F*_(1,65)_ = 1.399, *p* = 0.241, partial η^2^ = 0.021]. A *post-hoc*, pairwise, one-way ANCOVA with a factor of groups (adults who stutter and controls) was conducted for Mirror and Parallel conditions. The stuttering group performed a significantly fewer taps than the fluent controls in the Mirror task [*F*_(1,65)_ = 4.875, *p* = 0.031, partial η^2^ = 0.070] under an uncorrected statistical threshold (*p* = 0.05). The Parallel task showed a trend toward a significant difference [*F*_(1,65)_ = 3.883, *p* = 0.053, partial η^2^ = 0.056]. These *p*-values did not survive the adjusted statistical threshold after Bonferroni correction (*p* = 0.025). There were no significant correlations between percent stuttered syllables and performance in any condition (Mirror: *r* = −0.243, *p* = 0.159; Parallel: *r* = −0.245, *p* = 0.156).

**Table 3:** Tapping task.

	**Adults who stutter**	**Controls**	***p*** **-values**	**η^2^**
Mirror	114.1 ± 44.2	140.3 ± 37.8	*p* = 0.031	0.070
Parallel	61.2 ± 34.4	80.8 ± 24.5	*p* = 0.053	0.056

*Each shows the number of successful taps performed (mean ±SD) in 30 s*.

## Discussions

This study investigated bimanual coordination performance in adults who stutter and fluent controls. We found that the stuttering group performed worse than controls on tapping tasks only. In contrast, statistically significant differences in performance between groups were not evident for rotation tasks. Of the tasks used, tapping required control of bimanual movement timing and phase difference matching between hands. Conversely, in the rotation tasks, since both hands were connected during the performance and were not required to move independently, bimanual coordination control was required. Hence, these results support the theoretical framework, which states that stuttering is associated with a deficit in timing control. However, since the rotation tasks themselves were relatively difficult for both groups, we cannot conclude from this result alone, that motor dexterity is not different between adults who stutter and fluent speakers.

The two kinds of tasks used in this study differ in terms of speed of movement and their associated motor control demands. The rotational task requires careful movement of both hands and explicit, online monitoring of the motor state. Tapping, on the other hand, is a ballistic movement that requires precise timing control. In this sense, the rotation task may rely more on feedback control, while the tapping task may rely more on feed-forward control. Analogously, in speech production, the rapidity with which the respiratory, pharyngeal, laryngeal, and articulatory organs interact means that precise timing control of preprogrammed movements sequences is required to produce complex speech movements. With specific regard to stuttering, when the timing control is less demanding, such as is the case for slow, deliberate speech, where feedback can be employed to control accuracy, disfluencies are less evident (Andrews et al., 1982; Max et al., 2004). Depending on the rate, speech may rely more on feed-forward control, which is speed-oriented, or it may rely more on feedback control, which is accuracy-oriented (Anderson, 1975; Lammert et al., 2018). Thus, in a motor control sense, normal speech may be closer to the tapping task, while slow, deliberate speech may be closer to the rotation task.

Another possible explanation for our results is that the sensitivity to detect group differences may have been different between tasks. Although none of the group differences in the rotation tasks reached statistical significance, the averages of all conditions suggest an effect in the direction of worse performance for adults who stutter compared to controls (Figures 2, 3). Therefore, the difference in the presence/absence of statistical significance may be due to differences in the threshold for detecting significance, i.e., in the tapping task, the complexity or demands of the task exceeded the threshold for revealing a group difference, but that of rotation tasks did not. Therefore, experiments, where the number of rotation tasks, or complexity of hand movements is increased, should be considered in future research.

Similar speculation could apply to differences seen within the tapping task; in the *post-hoc* multiple comparisons, the Mirror task showed a significant difference (*p* = 0.031), while the Parallel task was marginally above the threshold for statistical significance (*p* = 0.053). In both groups, participants achieved approximately two times the number of performances in the Mirror compared with the Parallel task. This suggests the result is influenced by the statistical power to extract significant differences, since the mean of the stuttering group is skewed toward worse performance than that of the control group, even in the Parallel task. An increase in the duration of the task or the number of participants may reveal that a significant difference exists between groups in both conditions.

In previous studies, several bimanual tasks that required timing control have been used to investigate motor control characteristics in individuals who stutter. For example, Zelaznik et al. (1997) required participants to produce bimanual finger flexion and extension movements in time to a metronome. Their results showed that adults who stutter produced slower (lower peak velocity) and smaller amplitude finger movements compared with fluent controls. In addition, Zelaznik et al. (1997) reported that the stuttering group was more variable in maintaining a constant phase difference between the two effector fingers. This result is like the trend reported in the current study where we showed that individuals who stutter had relatively high variability (high SD) in the number of successful performances relative to fluent controls (Tables 1–3). Although there were no significant correlations between stuttering severity and task performance in any condition, this high variability may reflect the existence of heterogeneity among people who stutter. Similarly, when Webster (1990) required participants to produce a bimanual-asymmetrical tapping (tapping a key two times with one hand for each single tap of a key by the other hand), he showed that the tapping rates of the stuttering group were significantly slower than those of fluent group. When adults who stutter were required to write letters as quickly as possible bimanually, they were slower, made more mistakes, and formed poorer quality letters than fluent speakers (Webster, 1988). The studies by Webster used relatively complex bimanual tapping tasks and the results tend to show slower movement and poorer performance in people who stutter compared to fluent controls. On the other hand, the tapping tasks in our experiment were also not simple (Mirror and Parallel tapping), thus, they may have induced a significant difference in tapping rate between groups.

**Figure 5:**
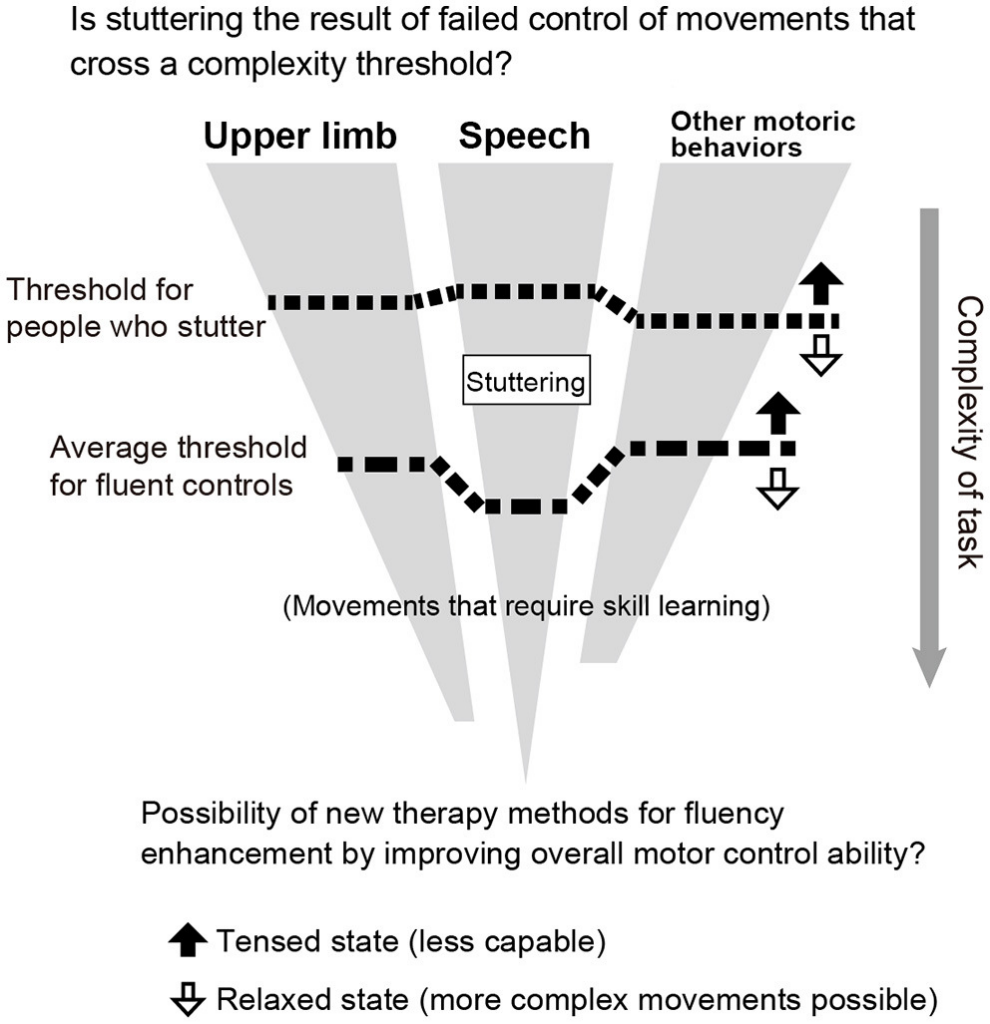
Relationship between stuttering and threshold of motor control ability (hypothesis).

As previous studies and the present study have shown, people who stutter perform worse than fluent speakers in motoric behaviors other than speech (at least for upper limb movements). This is especially true for tasks that require timing control. It is possible that stuttering is the result of an inability to control complex movements that cross a threshold of motor control ability (Figure 5). Since speech is the most complex motor act, stuttering speakers exhibit motor deficits explicitly only in speech production. However, when they are required to perform complex motor control tasks other than speech, their performance may be impaired. In addition, people who stutter are known to stutter more when they are in a state of tension. This may be because the threshold varies depending on the state of tension and relaxation (Figure 5, up and down arrows). In general, when we are tense, our movements are awkward, and when we are relaxed, we can perform more complex movements. If this hypothesis is true, then stuttering therapy may open the possibility of a new method for fluency enhancement training by improving overall motor control and emotion. Furthermore, the impairment of motor modalities other than speech suggests that the causative brain region in stuttering is not speech-specific but related to domain-general motor control structures, such as the basal ganglia, cerebellum, and supplementary motor area.

The basal ganglia play a key role in timing control in motor production and as discussed above, individuals who stutter have poorer behavioral performance on tasks where timing control is particularly important. Consistent with this, numerous studies have observed altered function and structure of the basal ganglia in stuttering participants compared with fluent controls (Wu et al., 1995; Lu et al., 2010b; Toyomura et al., 2011, 2015; Beal et al., 2013; Chang and Zhu, 2013; Foundas et al., 2013; Qiao et al., 2017; Sowman et al., 2017; Connally et al., 2018). However, there is no still consensus regarding the mechanism by which malfunction of the basal ganglia might cause stuttering; the difficulty in interpreting how particular basal ganglia dysfunction might manifest behaviorally relates to its complex structure and connections. An influential model of basal ganglia circuitry consists of three main loops referred to as the direct, indirect, and hyper-direct pathways (Alexander and Crutcher, 1990). Recently, in a sample of people who stutter, Metzger et al. (2018) reported that activity of the substantia nigra, one of the core basal ganglia substrates containing dopaminergic neurons that modulate striatal activity, correlated positively with stuttering severity. Furthermore, their study showed that adults who stutter exhibited altered network dynamics in the indirect pathway that passes through the external segment of the globus pallidus. This result implies that stuttering is associated with dopamine dysregulation and an imbalance between the direct and indirect pathways. Moreover, perturbations to a mathematical model of the basal ganglia that incorporates the direct and indirect pathways, have been shown to be able to simulate stuttering like disfluency (Civier et al., 2013).

Previous studies have shown the involvement of the cerebellum in stuttering, which is also involved in timing control, though the results are not necessarily consistent (e.g., De Nil et al., 2001, 2008; Watkins et al., 2008; Lu et al., 2010a; Ingham et al., 2012; Jiang et al., 2012; Toyomura et al., 2015; Sitek et al., 2016; Yang et al., 2016). There are four meta-analyses of neuroimaging on people who stutter, and of the four, three show significant involvement of the cerebellum (Brown et al., 2005; Budde et al., 2014; Belyk et al., 2015), though the most recent does not (Belyk et al., 2017). Howell et al. (2008) used the Dow-Moruzzi motor battery that includes bimanual tasks, which is like our experiment (Rotation 1 task), to investigate the cerebellar function of children who stutter. Speakers whose stuttering persisted beyond 12 years of age showed poorer performance compared with recovered speakers. On the contrary, our data of adults who stutter did not show a significant difference in the Rotation 1 task, which is most likely to tax cerebellar function. Differences in task complexity might explain these seemingly conflicting findings as there is a strong likelihood that localized brain dysfunctions can be compensated for up to a point; e.g., right inferior frontal gyrus overactivation in stuttering has been proposed to be compensatory in nature (Etchell et al., 2014a).

The supplementary motor area is also known to play an important role in complex movements, and hence, some authors have suggested that the supplementary motor area is associated with stuttering (e.g., Packman et al., 2007; Etchell et al., 2014b; Busan, 2020). Mirror and Parallel tapping have been heavily utilized in past human brain imaging to investigate the role of cortical and subcortical motor control, and many of these studies demonstrated significant involvement of the supplementary motor area. Since Parallel movement requires more complex and carefully coordinated movements than Mirror movement, the contrast between the two is suitable for extracting higher-order motor cortical representations of complex control. Many previous imaging studies, including those using positron emission tomography (PET; Sadato et al., 1997), functional magnetic resonance imaging (fMRI; Haslinger et al., 2004; Wu et al., 2010; Lin et al., 2017), and functional near-infrared spectroscopy (fNIRS; Wilson et al., 2014), have shown that the supplementary motor area is more activated during Parallel than during Mirror movement tasks. In an fMRI study by Wu et al. (2010) on PD, often highlighted in stuttering studies because of its shared features with stuttering (Alm, 2004), the control group showed higher activity in the supplementary motor area during Parallel movements compared with Mirror. The PD group had difficulty performing bimanual tasks and showed lower activity in the basal ganglia and supplementary motor area. Therefore, Parallel movements may be more associated with the supplementary motor area function than Mirror movements. Given that the supplementary motor area forms part of a basal ganglia-thalamocortical circuit, low performance in Parallel movements in adults who stutter may be linked to dysfunction of the supplementary motor area (Busan, 2020) and/or a basal ganglia-thalamocortical circuit (Metzger et al., 2018) that includes the supplementary motor area.

Non-invasive brain stimulation during bimanual movement, has also been used to investigate the role of the supplementary motor area. Stimulation of the area has been shown to modulate the performance of bimanual tasks in experiments using a transcranial magnetic stimulation (TMS; Serrien et al., 2002; Steyvers et al., 2003), transcranial direct current stimulation (tDCS; Carter et al., 2015), and transcranial alternating current stimulation (tACS; Miyaguchi et al., 2020). For example, repetitive TMS of the supplementary motor area at 5 Hz (Serrien et al., 2002) as well as at 20 Hz (Steyvers et al., 2003) reduced bimanual coupling during Parallel, but not during Mirror movements. Furthermore, when tDCS was applied to the supplementary motor area to increase its excitability, participants showed improved performance selectively for Parallel movements (Carter et al., 2015). On the whole, both brain imaging and stimulation studies suggest that the involvement of supplementary motor area is more significant for Parallel movements than Mirror movements. Therefore, our results showing that performance differs between people who stutter and fluent controls especially in Parallel conditions, suggest the involvement of the supplementary motor area (or a basal ganglia-thalamocortical circuit) in stuttering.

The brain regions reviewed in this study, the basal ganglia, cerebellum, and supplementary motor area, are all involved in general motor control and are not modality-specific. Although we cannot conclude whether the basal ganglia, cerebellum, supplementary motor area, or right inferior frontal gyrus (Wiener et al., 2010) is specifically involved in perturbed timing control in stuttering, based on our finding of significant difference only in the task requiring timing control (tapping tasks), we can at least claim that neural systems related to timing control are likely to be involved in the pathology of adulthood stuttering.

Timing control in motor implementation has also been examined from another perspective. Wing and Kristofferson (1973) propose an influential model that accounts for timing behavior in motor implementation. It has been used to infer the neural substrate of timing control in healthy participants as well as in patients with neurological disorders (e.g., Ivry et al., 1988; Franz et al., 1996; Bolbecker et al., 2011; Joundi et al., 2012). In their model, timing variance (interresponse interval variability) is assumed to be composed of the combined variance that arises from the internal clock (central time-keeping process) and that from motor implementation components (peripheral instability). Howell et al. (1997) showed that children who stutter have problems in the motor implementation component of timing. However, other studies on adults who stutter fail to corroborate this finding (Hulstijn et al., 1992; Max and Yudman, 2003). Observations of anomalous timing behavior in PD patients have been key to the inference that the substrate of timing is the basal ganglia (Meck et al., 2008; Joundi et al., 2012). The PD patients show timing deficits in simple rhythmic timing (O'Boyle et al., 1996; Harrington et al., 1998) and even in non-motor timing tasks such as interval estimation (Wild-Wall et al., 2008) and rhythm discrimination (Grahn and Brett, 2009), findings that implicate increased variance within internal clock as the deficits are not contingent on motor implementation. Similar impairments in simple rhythm production behavior are seen in stuttering (Olander et al., 2010; Falk et al., 2015). Furthermore, as is the case in PD, there is evidence to suggest this is not contingent on motor implementation *per se*, as passive neural entrainment to isochronous rhythms is altered in children who stutter compared with controls (Etchell et al., 2016).

Contrary views emerge from experiments on patients with cerebellar damage. For example, Ivry (1997) showed that, based on the Wing and Kristofferson model, lesions of the lateral cerebellum affect timing control whereas lesions of the medial cerebellum increase variance of motor implementation. Ivry (1997) and Ivry et al. (2002) posit models of timing in which the cerebellum has a central role in the regulation of temporal aspects of the movement. The implications of these models for stuttering are evident in the earliest neuroimaging studies of stuttering that, while somewhat inconsistent in their findings, strongly implicated cerebellar dysfunction as a core feature of stuttering (Wu et al., 1995; Fox et al., 1996, 2000; Ingham et al., 2000; De Nil et al., 2001, 2003; Toyomura et al., 2015).

There are some limitations to this study. We counted the number of times the participant correctly performed each task and did not take account of other elements of the movement. Previous studies using comparable methodologies have investigated kinematic movement elements such as peak velocity, movement duration, or peak velocity latency of the finger flexion/extension movements (Max et al., 2003), response initiation time, sequence execution time, or error rate of the sequence (Fox et al., 1996, 2000), and production finger tapping task (Forster and Webster, 2001). Significant group differences on such measures between stuttering and fluent groups have been reported. Including such measures as the number of discarded taps or analyzing synchronization timing error (Max and Yudman, 2003) in addition to the basic number of correct movements would possibly give a more nuanced view of the underlying mechanisms, which give rise to gross behavioral differences. We could not quantify kinematic features of the finger movements because of the video-based analysis. Furthermore, we did not collect information regarding musical expertise or gaming experience from participants. Given the effects on dexterity, such experiences might have, more careful characterization of manual skills should be considered in future studies.

In summary, this study found that adults who stutter perform worse in bimanual tapping tasks where both hands move independently and need timing control. However, in bimanual rotation tasks used to test motor dexterity, where both hands are connected, the performance of adults who stutter was not different from that of controls. These results support the theory that stuttering is associated with an abnormality of timing control.

## Data Availability Statement

The datasets presented in this article are not readily available because the specifications on data availability within the ethics approval. Requests to access the datasets should be directed to ak.toyomura@gmail.com.

## Ethics Statement

The studies involving human participants were reviewed and approved by Ethics committees of Tokyo Denki University and Gunma University. The patients/participants provided their written informed consent to participate in this study.

## Author Contributions

AT and TF designed the study and conducted the experiment. AT and research assistants analyzed the data. AT, TF, and PS discussed the results. AT and PS wrote the article. All authors contributed to the article and approved the submitted version.

## Funding

This study was supported by JSPS KAKENHI (Grant Numbers 24680025, 24650138, 16K00366, and 19H04195), the Mitsubishi Foundation, and the Strategic Research Project (07H012) for private universities from the Ministry of Education. PS is supported by the Australian Research Council (DE130100868, DP170103148, and DP 180102524) and the Australian Research Council Centre of Excellence for Cognition and its Disorders (http://www.ccd.edu.au; CE110001021).

## Conflict of Interest

The authors declare that the research was conducted in the absence of any commercial or financial relationships that could be construed as a potential conflict of interest.

## Publisher's Note

All claims expressed in this article are solely those of the authors and do not necessarily represent those of their affiliated organizations, or those of the publisher, the editors and the reviewers. Any product that may be evaluated in this article, or claim that may be made by its manufacturer, is not guaranteed or endorsed by the publisher.
